# The Public Health Governance of the COVID-19 Pandemic: A Bibliometric Analysis

**DOI:** 10.3390/healthcare10020299

**Published:** 2022-02-04

**Authors:** Keng Yang, Hanying Qi

**Affiliations:** 1 Institute of Economics, Tsinghua University, Beijing 100084, China; yangkengok@163.com; 2 One Belt-One Road Strategy Institute, Tsinghua University, Beijing 100084, China; 3 The New Type Key Think Tank of Zhejiang Province’s “Research Institute of Regulation and Public Policy”, Zhejiang University of Finance and Economics, Hangzhou 310018, China; 4 China Institute of Regulation Research, Zhejiang University of Finance and Economics, Hangzhou 310018, China

**Keywords:** public health emergency, COVID-19, public health governance, regulatory policies, bibliometric analysis

## Abstract

The 2019 global outbreak of COVID-19 has had a huge impact on public health governance systems around the world. In response, numerous scholars have conducted research on public health governance in the context of the COVID-19 pandemic. This paper provides a bibliometric analysis of 1437 documents retrieved from the Web of Science (WoS) core collection database, with 49,695 references. It analyses the research directions, countries of publications, core journals, leading authors and institutions and important publications. The paper also summarises research trends by analysing the co-occurrence of keywords, frequently cited documents and co-cited references. It summarises the global responses to COVID-19, including public health interventions and a range of supporting policies based on the features and impacts of the COVID-19 pandemic. The paper provides comprehensive literary support and clear lines of research for future studies on the governance or regulation of public health emergencies.

## 1. Introduction

In December 2019, a new coronavirus (SARS-CoV-2) emerged, triggering an outbreak of human acute respiratory syndrome centred in Wuhan, China [1]; this pandemic has become known as the 2019 coronavirus disease (COVID-19) pandemic. According to data from the World Health Organisation (WHO), by the end of October 2021, there were more than 200 million confirmed cases of COVID-19 worldwide, including more than 5 million deaths (available online: WHO Coronavirus (COVID-19) Dashboard, https://covid19.who.int/, date of access 1 December 2021). The COVID-19 pandemic has led to a massive global public health campaign, with government departments and public health agencies advocating increased hand washing, wearing masks in public places and social distancing to slow the spread of the virus. The outbreak of COVID-19 has highlighted the importance of having strong public health governance systems in place to safeguard public health.

Public health governance has a multifaceted role. For example, Helgesen (2014) argues that public health governance has two main roles: ‘health promotion’ and ‘disease prevention’ [2]. Carlson et al. (2015) defined six functions of public health governance: ‘policy development’, ‘resource stewardship’, ‘continuous improvement’, ’partner engagement’, ‘legal authority’ and ‘oversight of a health department’ [3]. In the case of infectious diseases, widespread prevalence will cause enormous economic and political damage to society. Effective public health governance can prevent and control this damage through health promotion, disease prevention and control, improved resource allocation, oversight of the public health sector and other activities. Thus, questions such as how to develop governance policies for catastrophic public health emergencies and which factors need to be taken into account deserve in-depth discussion and research.

In this period of intense scrutiny of disease prevention and control, how to take a long-term view, draw lessons from experience and build resilient public health systems have become hot topics of global concern. In the past two years, scholars around the world have published 1437 documents on public health governance during the COVID-19 pandemic; this is a clear indication of the importance of the field. Based on existing literature, we found that although there are many studies on public health governance policies for the COVID-19 pandemic, there are few articles that systematically analyse the overall lineage and direction of these studies. This article reviews the valuable experiences and research currently shared by scholars and institutions around the world. It provides a more comprehensive analysis for academics and practitioners to grasp the current status and shortcomings of public health governance during the COVID-19 pandemic. Furthermore, it also provides a useful exploration of further improvements to institutional mechanisms for catastrophic public health emergency governance and the public health regulatory system.

The following article consists of four parts in total. Section 2 presents the methodology and data. Section 3 provides an analysis of the distribution of the 1437 citing sources, includes the distribution of research direction, journals, countries or regions, institutions and authors. Section 4 is an analysis of research trends. Section 5 includes the discussion and conclusions.

## 2. Methodology and Data

### 2.1. Method

The methods used in this paper are bibliometric analysis and mapping of knowledge domains. Bibliometrics is a statistical and mathematical method to analyse the development of literature in a given field [4], helping researchers to understand current research trends, their distribution and core themes [5]. Mapping knowledge domains is a quantitative and visual research method that reveals knowledge about the structures of and connections and interactions between activities [4,6]. We used science mapping tools for the mapping analysis.

There are various mapping tools, including Bibliometrix, BibExcel, CitNetExplorer, HistCite, Leydesdorff Toolkit, SCI of SCI, Network Workbench, VOSviewer and CiteSpace [7,8]. We used VOSviewer (version 1.6.16) to execute our bibliometric analysis. VOSviewer was developed by van Eck and Waltman [7] at the Centre for Science and Technology Studies at Leiden University in the Netherlands, and it provides clear visualisation of knowledge mapping networks.

### 2.2. Data

The data were retrieved from the Web of Science (WoS) core collection database on 31 October 2021. From 1st January 2020 to 31 October 2021, 1437 documents have been published on the public health governance of COVID-19, with 49,695 references and a total of 12,959 citations. The 1437 documents that were retrieved from WoS can be recognized as citing sources. Their types include articles, editorial materials, letters, data papers, conferences, etc. According to the statistics of WoS, there were 1154 articles among them. The search query method mainly concerns the screening steps in Chen’s work [9]. That is, we combined multiple topical search queries to generate the data; the queries included keywords related to health public governance and COVID-19. Our search strategy was as follows:

(1) TS=(‘COVID19′ OR ‘COVID-19′ OR ‘COVID-2019′ OR ‘coronavirus disease 2019′ OR ‘SARS-CoV-2′ OR ‘sars2′ OR ‘2019-nCoV’ OR ‘2019 novel coronavirus’ OR ‘coronavirus disease 2019′ OR ‘coronavirus disease-19′ OR ‘novel coronavirus’ OR ‘SARS-CoV-2019′ OR ‘SARS-CoV-19′ OR ‘COVID’ OR ‘nCoV’)
(2) TS=(‘public health governance’ OR ‘public healthcare governance’ or ‘public health care governance’ or ‘global health governance’ or ‘public health polic*’ or ‘public healthcare polic*’ or ‘public health care polic*’) or TS=(‘public health’ NEAR governance) OR TS=(‘public healthcare’ NEAR governance) OR TS=(‘public health care’ NEAR governance) or TS=(governance NEAR ‘public health’) OR TS=(governance NEAR ‘public healthcare’) OR TS=(governance NEAR ‘public health care’) OR TS=(‘public healthcare’ NEAR polic*) OR TS=(‘public health care’ NEAR polic*) or TS=(‘public health’ NEAR polic*) or TS=(polic* NEAR ‘public health’) OR TS=(polic* NEAR ‘public healthcare’) OR TS=(polic* NEAR ‘public health care’)
(3) We combined the foregoing sets through the command (#1) AND (#2).

## 3. Bibliometric Analysis Results

### 3.1. Distribution of Research Directions

According to the WoS research directions, research related to public health governance during COVID-19 covers a total of 131 areas. We list the 10 areas with the highest number of publications below (see Table 1). The most productive research areas are public, environmental and occupational health (58.66%), followed by infectious diseases (57.97%), health care sciences services (46.49%) and the respiratory system (30.55%). The other areas in the top 10 are mainly related to social sciences and human behaviour, such as sociology, psychology, behavioural sciences, business economics, law and public administration. These areas also suggest that public health governance is closely related to socio-economic development, legal regulation, public administration and human psychological development.

### 3.2. Journal Distribution

According to WoS statistics, a total of 949 journals published COVID-19-related public health governance research. Table 2 reports the top 10 journals in terms of number of publications. The journal that has published the most papers is the *International Journal of Environmental Research and Public Health* (IJERPH). The main reason for its high number of publications is the journal’s focus on various aspects of public health topics and the journal’s efficiency in publishing articles. However, the average citation rate of publications is 7.9, which is not high compared to the top journals. This is due to the high volume of publications and the large number of articles published in 2021. Newly published papers are not yet able to receive much attention in a short period of time. Therefore, it is safe to assume that the productive journals have published many points of view and research contributions in the public health governance of COVID-19. *Healthcare*, which has the same publisher (Multidisciplinary Digital Publishing Institute, or MDPI, available online: https://www.mdpi.com/, accessed on 1 December 2021) as IJERPH, also has a high number of articles, ranking seventh among the top 10 journals. The second most published journal is *Frontiers in Public Health*, followed by *PLOS One*, *Journal of Medical Internet Research* and *Public Health*.

Citations can lend insight into the influence or popularity of an article, and the top 10 journals in terms of total citations were thus further analysed (see Table 3). The most cited journals were *JAMA—Journal of the American Medical Association*, followed by *Annals of Internal Medicine* and *Lancet*. These journals are the top journals in their respective disciplines. Papers published in these journals received great attention during the time period covered by this paper.

The most cited paper in *JAMA—Journal of the American Medical Association* is from Pan et al. (2020) and is entitled ‘Association of public health interventions with the epidemiology of the COVID-19 outbreak in Wuhan, China’ (citations = 621). This paper quantifies the relationship between public health interventions and prevention and control of the COVID-19 pandemic in China [10]. The most cited paper in *Annals of Internal Medicine* is ‘Diagnostic testing for severe acute respiratory syndrome-related coronavirus 2: A narrative review’ (citations = 277). This article reviews research on the global disparity in diagnostic testing capabilities and is a study of public policy approach [11]. The paper with the highest number of citations in *The Lancet* is ‘Dementia prevention, intervention, and care: 2020 report of the Lancet Commission’. This article, written by the Lancet Commission, reports on the impact of the COVID-19 pandemic on a specific group of people with dementia and suggests appropriate public health precautions [12].

### 3.3. The Distribution of Countries/Regions

Table 4 reports the top 10 countries or regions in terms of their number of publications. The highest number of articles were published by scholars from the USA, with 561 publications in two years. The second most productive country is England, and China is third with 170 publications. Of the 10 countries, seven are developed, while three (China, Brazil and India) are emerging market countries. Scholars from these 10 countries have made relatively large contributions to public health governance research during the COVID-19 pandemic.

Furthermore, we used VOSviewer to build a collaborative network between countries. The network is mainly composed of countries that published more than one article, with 111 nodes in total. The size of the nodes indicates the number of publications, the colour of the nodes represents the clusters (16 clusters) and the thickness of the lines between the nodes represents collaboration strength. The cluster categories are mainly implemented by VOSviewer based on association strength.

**Figure 1:**
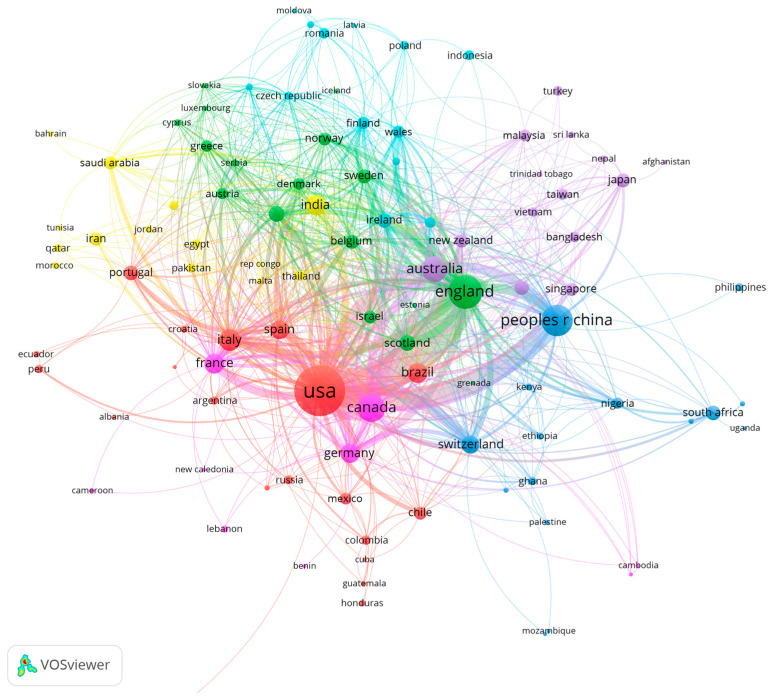
The co-authorship network of countries/regions.

According to Figure 1, the 10 countries in Table 4 are core nodes in their respective categories. Scholars from the USA have strong collaborative partnerships with scholars from China, Canada, France, Australia and Switzerland. Scholars from England have extensive collaborations with scholars from Scotland, Germany and Switzerland. China has strong collaborative partnerships with developed countries such as Australia, the USA and Canada. In terms of the link strength, the strongest and most collaborative relationships are between the USA and China.

### 3.4. The Distribution of Institutions

This section presents a statistical analysis of the author affiliations of the 1437 citing sources and lists the top 10 productive institutions. The University of London is the most prolific publisher with 64 documents, and it has the second highest citation rate after Harvard University. Harvard University is the second most productive institution, followed by the University of California System. Among the top 10 institutions, most are from the USA (five schools or university systems).

**Figure 2:**
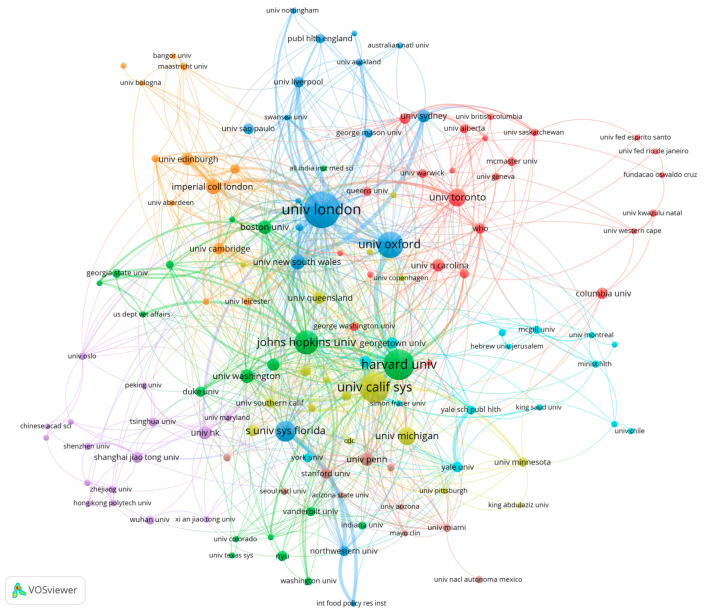
The co-authorship network of institutions. Note(s): The colours refer to cluster, node size refers to publication number and line thickness refers to cooperative strength.

We also carried out an in-depth analysis of institutional co-authorship. We used VOSviewer to construct a collaboration network. The network consisted of 134 institutions with five or more articles and was divided into 12 clusters based on association strength. The network map is shown in Figure 2. As shown, the node of University of London is the largest and is at the core of each linkage. Combined with Table 5, it has the strongest total link strength, followed by Harvard University and the University of California System. This suggests that academics have played an important role in the area of public health governance during the COVID-19 pandemic.

The collaborative networks in Figure 2 show strong geographical clustering. For example, the University of London, which has the highest number of publications, has stronger collaborations with institutions mainly from the UK, such as the University of Oxford, Imperial College London, University New South Wales and so on. The green and yellow clusters are more likely to be US-based institutions, with thicker link lines within these clusters that indicate stronger collaborative relationships. Among the core nodes, there is a strong partnership between Harvard University and the University of Oxford.

### 3.5. Author Distribution

**Figure 3:**
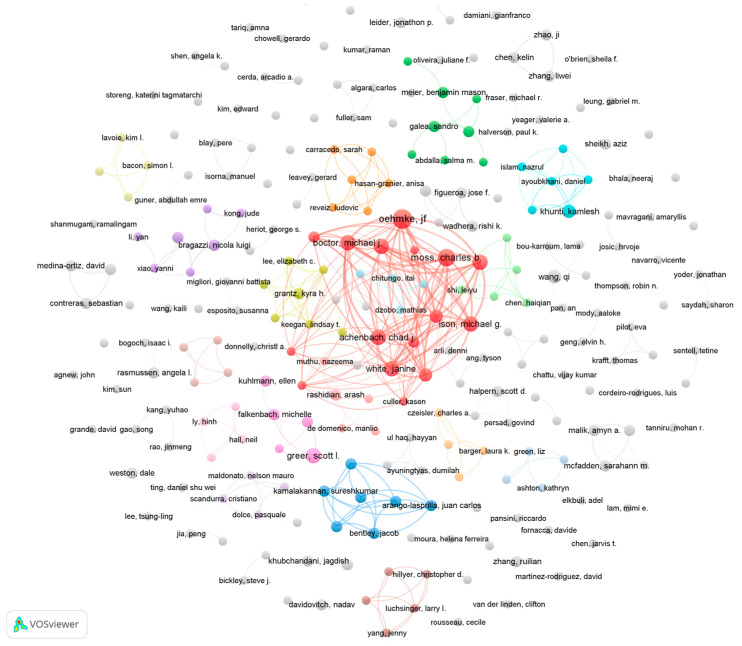
The co-authorship network of authors (publications). Note(s): Node size represents the number of publications. Node colour refers to clusters. The links refer to co-authorship.

**Figure 4:**
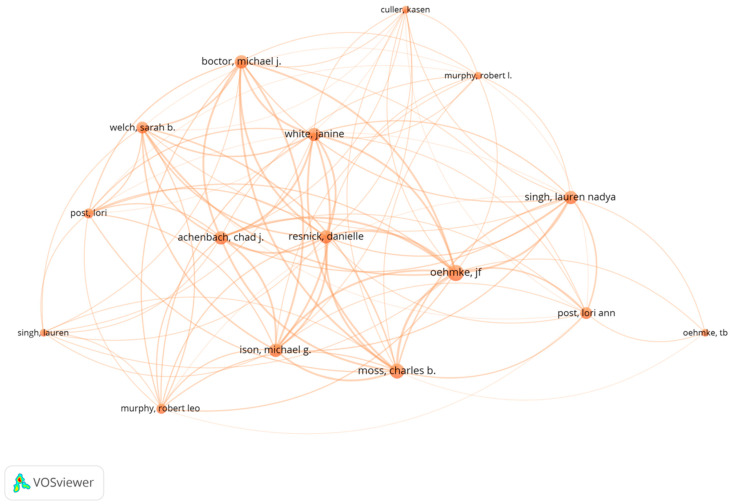
The co-authorship network (this is based on citations).

Table 6 reports the top 10 productive authors. These authors have an average of around five publications and have a relatively similar total link strength. According to Figure 3 and Figure 4, there is a strong collaborative relationship between the eight authors in Table 6. With the exception of Greer and Khunti, the remaining eight authors have co-authored five papers, as reported by the WoS citation report.

Most of these papers were published in 2021 and focus on monitoring the transmission characteristics of COVID-19 around the world, including in Central Asia [13], the USA [14], Canada [15], Europe [16], the Middle East and South Africa [17]. All five papers apply the dynamic panel data (DPD) model approach proposed by Oehmke et al. (2020). The DPD model was mainly used to derive surveillance metrics [18]. These dynamic surveillance indicators can provide an important factual basis for public health policy regarding COVID-19.

Figure 3 shows a visualisation of the author collaboration network. The figure includes 253 authors with two or more publications. The most dominant collaborative network in the graph consists of the eight authors in Table 6. The remainder of the authors present multiple collaborative teams, although these teams produced a relatively low number of publications.

**Figure 5:**
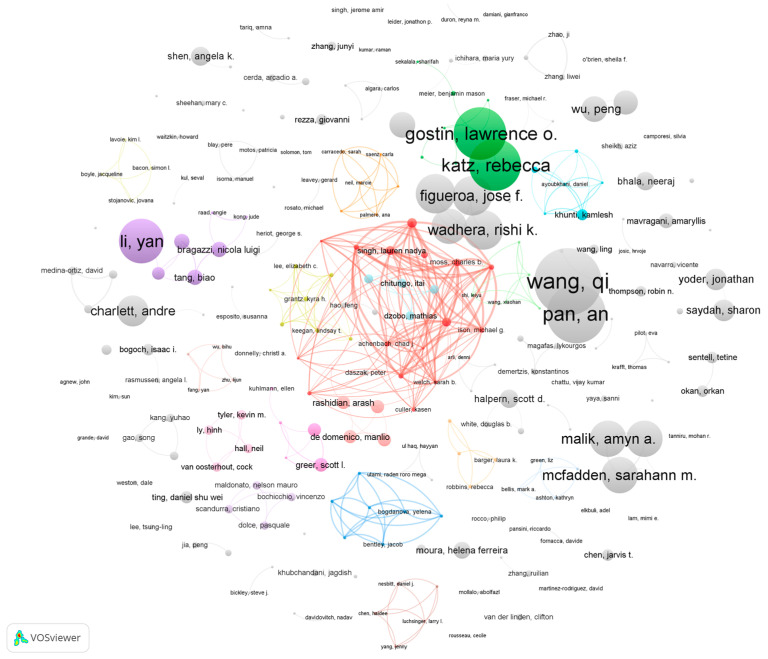
The co-authorship network of authors (citations). Note(s): The node size represents the frequency of citations. The colours of nodes refer to clusters. The links refer to co-authorship.

The network composition in Figure 5 is the same as in Figure 3. The only difference between the two is that the node sizes indicate different meanings. Node size in Figure 5 represents the total number of publication citations for an author’s publications. The core authors in Figure 5 are mainly Qi Wang, An Pan, L. Gostin, R. Katz, Yan Li and others. Although these authors have published fewer articles, their papers have had a greater impact. Among these highly cited authors, we investigated the h-index (see Table 7) and found that Qi Wang’s research during the COVID-19 pandemic may have had the highest impaction in the fields of COVID-19 public health governance.

## 4. Research Trends

### 4.1. The Co-Occurrence of Keywords

**Figure 6:**
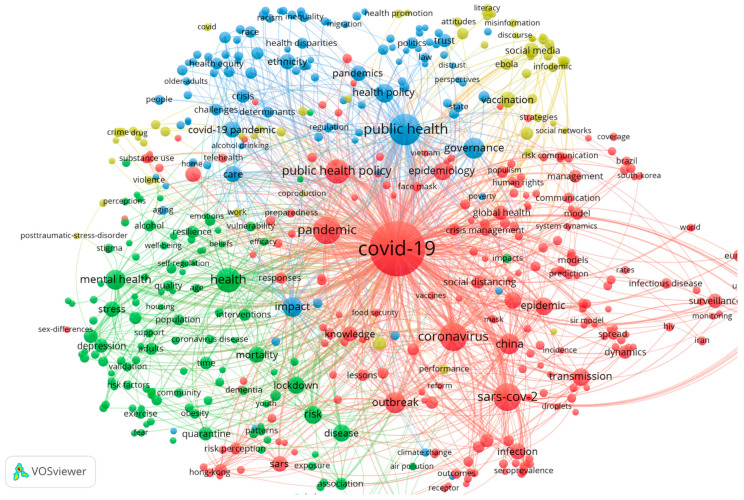
The co-occurrence network of keywords. Note(s): Node size represents the frequency; the colours represent the clusters.

**Figure 7:**
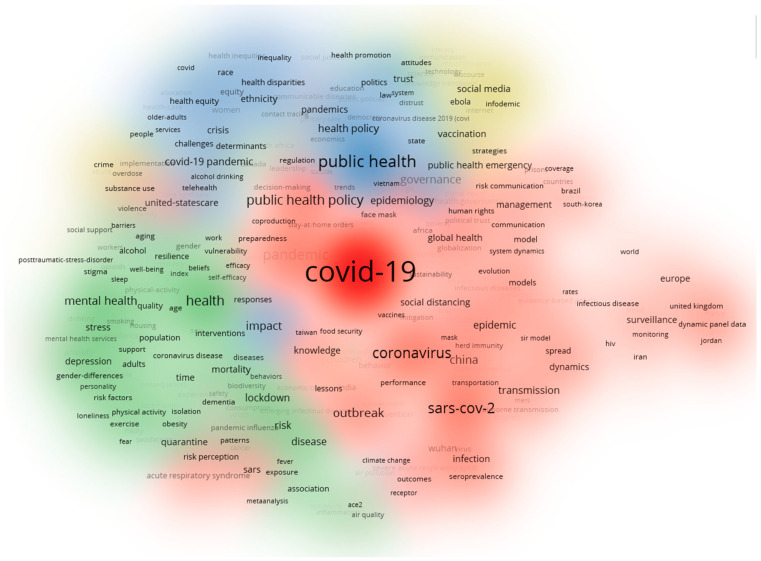
The cluster density visualization of the keywords’ co-occurrence network.

The total number of keywords is 4088. We used VOSviewer to construct a network of keyword co-occurrence. The minimum number of occurrences of a keyword in Figure 6 is three. There are 519 nodes and four clusters in the network map. These clusters were also shown in Figure 7. According to Figure 6 and Figure 7, there are large nodes in each cluster. The largest node is COVID-19, with all clusters co-occurring with this keyword. This is largely because the focus of this paper is on public health emergency governance during the COVID-19 pandemic. The main nodes of each cluster are discussed in detail below.

(1) The red cluster. The core keywords are ‘COVID-19′, ‘pandemic’, ‘public health policy’, ‘coronavirus’, ‘influenza’, ‘epidemic’, ‘epidemiology’, ‘outbreak’, ‘transmission’, ‘surveillance’, ‘crisis management’ and so on. This clustering focuses on the spread and transmission of COVID-19. For public health authorities, knowledge of the transmission characteristics of pandemic viruses is crucial for managing catastrophic public health emergencies. For example, monitoring the transmission characteristics of COVID-19 in different countries allows targeted public policies to be developed. Overall, this clustering suggests that one of the research fields on the public health governance of COVID-19 pandemic is the study of the epidemiological characteristics of COVID-19 outbreaks. This study has significant crossover with disciplines such as epidemiology and infectious diseases.
(2) The green cluster. The main keywords in this cluster include ‘health’, ‘mental health’, ‘stress’, ‘mortality’, ‘lockdown’, ‘risk’, ‘disease’ and so forth. These keywords are closely associated with COVID-19. This category suggests that the literature also focuses on the social impact of COVID-19, including the impact on people’s mental health, mortality, underlying disease and so on. This indicates that public health governance also needs to delve into and capture the impact effects of infectious diseases.
(3) The blue cluster. The crucial keywords include ‘public health’, ‘governance’, ‘regulation’ and ‘health policy’. These main keywords are also associated with ‘heath disparities’, ‘equality’, ‘race’, ‘inequality’ and so on. This part of the study focuses on the public health regulator polity and the relationship between public health policy and other health issues, such as health equity.
(4) The yellow cluster. The keywords in this cluster include ‘public health emergency’, ‘Ebola’, ‘Internet’, ‘infodemic’, ‘social media’, ‘vaccination’, ‘attitudes’, ‘literacy’ and similar terms. The literature related to this cluster examines the relationship between digital technologies such as the Internet, online media and COVID-19 or the use of digital technologies in public health governance of COVID-19. For example, one study in May 2020 used an online platform to investigate perceived risk of the COVID-19 pandemic, acceptance of the COVID-19 vaccine and trust in information sources among US adults [19].

**Figure 8:**
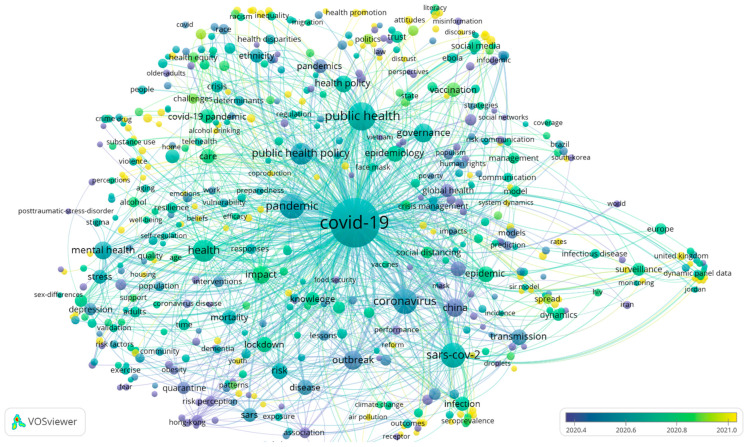
The overlay visualisation of the keywords co-occurrence network. Note(s): Colour represents the average occurrence time of a keyword; node size represents the frequency.

Additionally, we plotted the overlay visualisation of keyword co-occurrence (see Figure 8). The darkest colours appear earliest. The earliest occurrences correspond to the red clusters in Figure 7, thereby indicating that early scholars focused on the characteristics of COVID-19. The other clusters are relatively lighter in colour. The areas corresponding to the blue and yellow clusters in Figure 7 are the keywords that appear in literature published near November 2021, indicating that the overall research focus is expanding towards policy as well as digital technology as the COVID-19 pandemic progresses.

### 4.2. Document Citation Numbers

Table 8 reports a total of 23 papers with over 100 citations from the 1437 documents cited. These papers reflect research themes that have received extensive attention within public health governance of COVID-19. The themes of these 23 papers focus on the following areas.

(1) Studies of COVID-19 epidemiological features.
  (a) Features: Some scholars have studied the characteristics of regional disparities in diagnostic testing rates, mortality rates and hospitalisation rates in the context of the COVID-19 pandemic outbreak [20]. Some studies have analysed differences in infection and mortality rates by age group [21] or the duration of different symptoms and time taken to recover [22]. These studies can provide empirical evidence for public policies to mitigate the spread of novel coronaviruses.
  (b) Features and public health policy: Based on study of the infectious features of Canadian cases, Bullard et al. (2020) recommended isolation from the community for at least 10 days after the onset of symptoms of COVID-19 infections [23]. This study helped to accurately identify the period of maximum risk of COVID-19 transmission and provided empirical evidence for public health policies to rapidly interrupt the virus transmission chain [23]. Singanayagam et al. (2020) reached the same conclusion from a study of infection cases in the UK. They also suggested that asymptomatic infected persons may be a source of virus transmission [24]. The transmission features of COVID-19 cases in Hong Kong have also been studied. It was found that isolation has limited effectiveness in reducing transmission if started only after diagnosis because the virus spreads before the onset of symptoms, and there is a delay between symptom onset and diagnosis [25]; this lends support for public policies such as crowd reduction and social distancing. There is also research into the transmission features of COVID-19 in the child population to provide empirical evidence on whether to open schools [26].
(2) Public health interventions for controlling the transmission of COVID-19 have included diagnostic testing [11,27]; intensive intracity and intercity traffic restrictions, social distancing, home isolation and centralised quarantines, and improvement of medical resources [10]; improvement of COVID-19 vaccine acceptance [19].
(3) Public health governance policies for special groups: During the COVID-19 pandemic, people with dementia have been at greater risk of infection and death. As such, studies have recommended stricter public health protection measures, such as restricting movement and increasing diagnostic testing of caregivers [12]. Cancer patients have experienced risk of delayed management, exposure to infection during care and limited allocation of healthcare resources during the COVID-19 pandemic. To combat these issues, the use of digital technology platforms to provide telemedicine treatments and educational guidance has become an effective public policy for cancer patients [28]. For people with addiction disorders, COVID-19 increases the severity of the disorder [29]. There are also especially susceptible occupational groups, such as workers in meat processing plants and health care workers, who have a higher risk of being infected with COVID-19 [30,31]. These groups need to be supported by specific public services.
(4) Public health regulatory policies for social issues related to the COVID-19 pandemic: The two main social issues impacted by COVID-19 are mental health and health inequalities. In terms of mental health, a study on the psychological stress of Americans during the COVID-19 pandemic found that the pandemic could cause significant psychological stress and anxiety. Specific public health interventions should consider mental health interventions and provide more social support for the psychologically vulnerable [32]. In terms of health inequalities, there are economic, ethnic and geographical inequalities in COVID-19 infection and mortality rates [33]. These health inequalities are often influenced by biological and socioeconomic factors [33]. Current practices of social distancing and isolation regulatory policies may also contribute to health inequalities. In particular, the economic crisis associated with the COVID-19 pandemic will further increase health inequalities [33]; in response, policies for public health emergencies need to consider long-term responses to addressing inequalities in the post-pandemic era.
(5) Evaluation of the effectiveness of public health governance: In the early days of the COVID-19 pandemic, non-pharmacological interventions such as social distancing were evaluated, and concerns were expressed about the possible effects of these interventions. Some scholars have argued that involuntary restrictions on movement may result in a loss of social welfare, such as mistrust of government, loss of economic resources and possibly even violations of human rights, such as the right to dignity, privacy and freedom of movement [34]. Hence, they suggested that public health emergency regulatory interventions should proceed as follows: fully characterising COVID-19, conducting intensive epidemiological surveys, rapidly proposing drug responses and fully mobilising supply chain support for material needs [34]. Based on early infectious disease cases, Pan An et al. (2020) provided an assessment of the pandemic control effects of various public health interventions in China [10]. Their results showed that movement restrictions can significantly reduce the rate of virus transmission, rapid diagnosis can reduce aggregated transmission in households and infection rates for health care workers were very high during transmission outbreaks, making health care worker protection policies especially important. Similarly, Badr et al. (2020) studied the relationship between social distancing and COVID-19 transmission in the United States and found that social distancing policies were effective in reducing transmission [35].
(6) Studies on other features of COVID-19 pandemic-covered topics including the impact of the COVID-19 pandemic on people’s travel patterns [36], lifestyles and dietary habits [37]; these impacts have led to corresponding public health policies.

**Table 8:** Articles with more than 100 citations.

Code	Title	Authors	Citations
1	Association of Public Health Interventions with the Epidemiology of the COVID-19 Outbreak in Wuhan, China	Pan An, et al. [10]	621
2	The Novel Coronavirus Originating in Wuhan, China Challenges for Global Health Governance	Phelan, Alexandra L., et al. [34]	512
3	Dementia prevention, intervention, and care: 2020 report of the Lancet Commission	Livingston, G., et al. [12]	502
4	Predicting Infectious Severe Acute Respiratory Syndrome Coronavirus 2 From Diagnostic Samples	Bullard, J., et al. [23]	381
5	Diagnostic Testing for Severe Acute Respiratory Syndrome-Related Coronavirus 2: A Narrative Review	Cheng, Matthew P., et al. [11]	277
6	Variation in COVID-19 Hospitalizations and Deaths Across New York City Boroughs	Wadhera, Rishi K., et al. [20]	267
7	Symptom Duration and Risk Factors for Delayed Return to Usual Health Among Outpatients with COVID-19 in a Multistate Health Care Systems Network—United States, March–June 2020	Tenforde, Mark W., et al. [22]	221
8	Duration of infectiousness and correlation with RT-PCR cycle threshold values in cases of COVID-19, England, January to May 2020	Singanayagam, A., et al. [24]	214
9	Determinants of COVID-19 vaccine acceptance in the US	Malik, Amyn A., et al. [19]	211
10	Association between mobility patterns and COVID-19 transmission in the USA: a mathematical modelling study	Badr, Hamada S., et al. [35]	189
11	A War on Two Fronts: Cancer Care in the Time of COVID-19	Kutikov, A., et al. [28]	173
12	Americans’ COVID-19 Stress, Coping, and Adherence to CDC Guidelines	Park, Crystal L., et al. [32]	150
13	Clustering and superspreading potential of SARS-CoV-2 infections in Hong Kong	Adam, Dillon C., et al. [25]	143
14	The COVID-19 pandemic and health inequalities	Bambra, C., et al. [33]	142
15	COVID-19: potential effects on Chinese citizens’ lifestyle and travel	Wen, J., et al. [36]	136
16	COVID-19 Among Workers in Meat and Poultry Processing Facilities—19 States, April 2020	Dyal, Jonathan W., et al. [30]	122
17	Assessing the age specificity of infection fatality rates for COVID-19: systematic review, meta-analysis, and public policy implications	Levin, Andrew T., et al. [21]	115
18	Transmission of SARS-CoV-2 in Australian educational settings: a prospective cohort study	Macartney, K., et al. [26]	114
19	COVID-19 Confinement and Changes of Adolescent’s Dietary Trends in Italy, Spain, Chile, Colombia and Brazil	Belen Ruiz-Roso, M., et al. [37]	111
20	Mitigating and learning from the impact of COVID-19 infection on addictive disorders	Marsden, J., et al. [29]	108
21	SARS-CoV-2-Positive Sputum and Feces After Conversion of Pharyngeal Samples in Patients With COVID-19	Chen, Chen, et al. [38]	107
22	Diagnostic Testing for the Novel Coronavirus	Sharfstein, Joshua M., et al. [27]	107
23	Healthcare workers & SARS-CoV-2 infection in India: A case-control investigation in the time of COVID-19	Chatterjee, P., et al. [31]	103

### 4.3. The Co-Citation of Cited References

**Figure 9:**
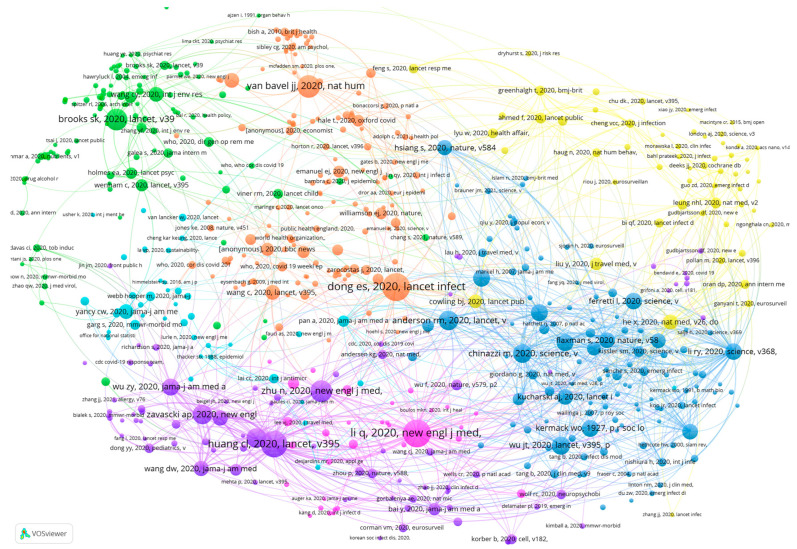
Co-citation network of cited references. Note(s): Node size refers to the citations; colour refers to the clusters.

Co-cited references provide theoretical and empirical support for the citing documents. We therefore constructed a co-citation network of references based on the citing sources. According to VOSviewer, there were 49,695 references in 1437 citing sources. We selected references with more than five citations as the nodes of the co-citation network, as shown in Figure 9. There are 596 nodes in the figure, which are divided into six clusters. Each of these clusters has core nodes that are larger in size and links. We summarised these nodes according to the clusters and analysed the ways in which these provided support for the citing sources.

The two most notable core references in the orange cluster are Dong et al. (2020) and Bavel et al. (2020). Dong et al. (2020) introduced a real-time tracking system for COVID-19 cases that can provide public health departments as well as the public with real-time information on the development of the pandemic [39]. Bavel et al. (2020) summarised the responses and behaviours of individuals, groups, governments and countries to the COVID-19 pandemic from a social and behavioural sciences perspective to provide support for future policy formulation in public health emergency governance [40]. Such research provides important support for the development of public policies from the technical, social and behavioural perspectives.

The core references in the green cluster are mainly concerned with mental health. Brooks et al. (2020) reviewed research on the impact of segregation as a public health policy on people’s mental health. Through their literature review, they found that isolation can exacerbate people’s psychological stress. Stressors include financial loss, lack of information and frustration. In this regard, public health institutions should isolate infected persons for clear reasons and for no longer than the required period of isolation [41]. Research also suggested that accurate health information about the COVID-19 pandemic and the promotion of special precautionary measures could effectively reduce negative impacts on mental health [42].

The references in the purple cluster focus on the clinical features of COVID-19. The most cited reference was published by Huang et al. on 24 January 2020. This article analysed the age distribution and symptom presentation of 41 patients with COVID-19 based on laboratory data [43]. Zhu et al. (2020) also published a study on the clinical features of COVID-19 in the same period [44]. Additionally, there were discussions of hallmark symptoms at the onset of COVID-19 infections [45,46]. These are early papers on the features of 2019 coronavirus infections and provide an empirical basis for subsequent studies on epidemiological features.

The blue cluster focuses on issues related to the transmission characteristics of COVID-19. Anderson et al. (2020) examined whether the introduction of national measures such as quarantine were effective in stopping the transmission of COVID-19 [47]. Similarly, Chinazzi et al. (2020) studied the impact of travel restrictions on national and international transmission of COVID-19 [48]. Joseph used the Susceptible–Exposed–Infectious–Recovered (SEIR) COVID-19 transmission model to simulate and predict the size and spread of COVID-19 pneumonia [49].

The nodes in the yellow cluster are overall less frequently cited than the other clusters, and the core nodes are smaller in size. The most notable of these references are He et al. (2020) and Cowling et al. (2020); the former studied the transmission characteristics of COVID-19 infections before the onset of symptoms, or pre-symptomatic transmission [50], while the latter studied the mitigating effects of public policies such as isolation and social distancing on the spread of the virus [51]. The study topics of this cluster are more similar to that of the blue cluster.

## 5. Discussion and Conclusions

### 5.1. Discussion

**Figure 10:**
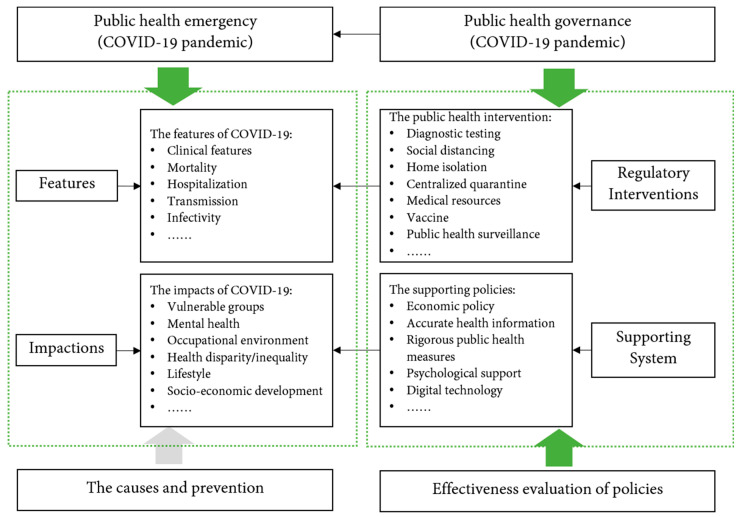
An analytical framework for research on public health governance during the COVID-19 pandemic.

We used an analytical framework to summarise the research directions and trends of public health governance of COVID-19 (see Figure 10). The aim of the framework is to provide a more intuitive research overview for scholars and to support future research on the governance of catastrophic public health emergencies.

Figure 10 shows that the focus of research on public health governance during the COVID-19 pandemic encompasses three aspects. The first is to study the features of COVID-19 and its effects. In catastrophic public health emergencies, the most important aim of public health governance institutions or researchers is to use technology and scientific experiments to study the epidemiological features and impact effects of the emergency itself. The second is to study and propose public health governance regulatory interventions and supporting systems for COVID-19 that can be used to regulate or eliminate public health risks. The third is to evaluate the effects of the policies. By evaluating and comparing policy effects, more appropriate public health governance policies will be promoted to improve the efficiency of public health governance system. These three aspects follow the basic paradigm of public health governance, namely the discovery-to-control paradigm. Existing research on public health governance has focused on providing effective responses and regulatory policies at each stage of the discovery-to-control process.

However, at the beginning of the 21st century, some scholars pointed out that a simple discovery-to-control paradigm would require huge governance costs and cause resource scarcity problems [52]. Neubauer (2005) argued that the discovery-to-control governance paradigm should be shifted to a ‘research-to-prevention-to-discovery-to-control’ paradigm. [52]. Prevention is also an important element of public health governance, and scholars and institutions in various countries have been making efforts in this regard by exploring the causes of epidemics, the history of the emergence of coronaviruses and so on [53,54]. However, there are fewer studies examining the causes and prevention of infectious diseases such as COVID-19 from a public health governance perspective (see Figure 9). This is an area that deserves further exploration. At the same time, public health governance also involves multifaceted institutional support, including a coordinated and effective public health governance system, professional staffing, special clinical systems and public infrastructure systems. These are also areas where public health governance needs further improvement and research.

### 5.2. Conclusions

In this paper, we used VOSviewer and Excel to analyse the distribution of publications and research trends on public health governance during the COVID-19 pandemic. We advance three main conclusions.

First, we summarised the main research directions in the field of public health governance related to the COVID-19 pandemic. The research direction that has attracted the most publications is “public, environmental and occupational health”.

Second, we identified the journals, countries (or regions), institutions and authors that have made major contributions to the field. The journal with the most publications was the *International Journal of Environmental Research and Public Health*, while *JAMA—Journal of the American Medical Association* had the highest number of average citations. The country with the most articles was the United States. The institution with the most publications was the University of London. The most influential authors were Wang Qi, Pan An, Gostin, Katz, Li Yan.

Third, we identified research trends in public health governance during COVID-19. These trends include the features and impacts of COVID-19, interventions and supporting policies, and the effectiveness of the policies.

This paper has important implications for researchers and regulators in understanding how the public health sector responds to public health emergencies. The summary in Figure 9 shows the elements that should be taken into consideration to develop and implement public health policies. It also provides empirical support for future research on improving public health governance systems and helps public health researchers to understand possible future research directions.

This paper has several limitations. The WoS Core Collection was used as the data source to obtain high quality research, which may lead to a significant amount of literature being excluded, such as the literature from Scopus, PubMed/Medline and others. There could be other bibliometric analyses of other databases in the future. Furthermore, as public health governance is a global topic and varies from country to country, the content of the research may also vary. In the future, country samples can be selected to conduct comparative studies.

## Figures and Tables

**Table 1:** Top 10 research directions.

Direction	Publications	Percentage (%)
Public, Environmental and Occupational Health	843	58.66
Infectious Diseases	833	57.97
Health Care Sciences Services	668	46.49
Respiratory System	439	30.55
Sociology	307	21.36
Psychology	296	20.60
Behavioural Sciences	241	16.77
Business Economics	238	16.56
Government Law	219	15.24
Public Administration	183	12.73

Note(s): The data were retrieved from WoS. All tables in the text have the same data source unless stated otherwise. The total number of publications (N = 1437) was used to calculate the percentage of publications.

**Table 2:** Top 10 most productive journals.

Journal	Publications	Percentage (%)	Citations	Avg. Citation	IF-5 Years
International Journal of Environmental Research and Public Health	56	3.90	445	7.9	3.79
Frontiers in Public Health	51	3.55	129	2.5	4.02
PLOS One	39	2.71	185	4.7	3.79
Journal of Medical Internet Research	23	1.60	173	7.5	7.26
Public Health	20	1.39	75	3.8	2.81
BMC Public Health	15	1.04	51	3.4	4.00
Healthcare	12	0.84	20	1.6	3.04
Frontiers in Psychology	11	0.77	97	8.8	3.62
Global Public Health	11	0.77	145	13.1	2.67
BMJ Open	10	0.70	14	1.4	3.42

Note(s): The total number of publications (N = 1437) was used to calculate the percentage of publications. Avg. is the abbreviation for average.

**Table 3:** Top 10 journals with high citations.

Journal	Publications	Citations	Avg. Citation	IF-5 Years
JAMA—Journal of the American Medical Association	7	1539	219.86	60.15
Annals of Internal Medicine	8	750	93.75	25.27
The Lancet	6	628	104.67	77.24
MMWR—Morbidity and Mortality Weekly Report	9	524	58.22	12.99
International Journal of Environmental Research and Public Health	56	445	7.9	3.79
Clinical Infectious Diseases	2	402	201	9.60
eClinicalMedicine	4	360	90	N/A
Lancet Infectious Diseases	5	268	53.6	24.91
Eurosurveillance	2	246	123	6.02
Nutrients	6	239	39.8	6.35

**Table 4:** The top 10 productive countries/region.

Country/Region	Publications	Percentage (%)
USA	561	39.013
England	200	13.908
China	170	11.822
Canada	128	8.901
Australia	99	6.885
Italy	66	4.59
India	56	3.894
France	54	3.755
Brazil	53	3.686
Germany	48	3.338

**Table 5:** Top 10 productive institutions.

Institution	Documents	Citations	Total Link Strength
University of London	64	1193	382
Harvard University	51	2020	222
University of California System	50	889	188
University of Oxford	41	554	170
Johns Hopkins University	36	1180	165
State University System of Florida	29	231	96
University of Michigan	24	81	48
University of Toronto	24	236	100
Imperial College London	19	142	83
University of New South Wales	18	344	100

Note(s): These results were calculated by VOS based on data retrieved from WoS.

**Table 6:** The top 10 productive authors.

Author	Documents	Citations	H-Index	Total Link Strength
Oehmke, JF	7	35	4	58
Moss, Charles B.	6	24	3	55
Achenbach, Chad J.	5	14	2	51
Boctor, Michael J.	5	14	2	51
Greer, Scott L.	5	60	2	6
Ison, Michael G.	5	14	2	51
Resnick, Danielle	5	14	2	51
Singh, Lauren N.	5	31	3	38
White, Janine	5	14	2	51
Khunti, Kamlesh	4	44	3	9

Note(s): These results were calculated by VOSviewer based on WoS data.

**Table 7:** The top five authors with highest citation.

Author	Total Link Strength	Documents	Citations	H-Index
Qi Wang	2	4	736	4
An Pan	1	2	594	1
Lawrence O Gostin	4	3	496	3
Rebecca Katz	1	2	486	2
Yan Li	1	2	383	2

## Data Availability

No data statement.
